# Bio-stimulants for plant growth promotion and sustainable management of *Rhizoctonia Solani* causing black scurf of potato tubers

**DOI:** 10.1186/s12870-025-07689-y

**Published:** 2025-12-02

**Authors:** Sozan Eid El-Abeid, Eman Said Elshewy, Ayman Y. Ahmed

**Affiliations:** 1 Nanotechnology & Advanced Nano-Materials Laboratory (NANML), Mycology and Disease Survey Research Department, Plant Pathology Research Institute, Agricultural Research Center, Giza, 12619 Egypt; 2 Department of Vegetable Diseases Research, (PPRI), (ARC), Giza, Egypt

**Keywords:** Bio-stimulants, Potato, Black scurf, Indole-3-acetic acid, Quality of products, Starch, Α-amylase contents

## Abstract

**Background:**

Bio-stimulants are natural substances that have achieved considerable advances. However, they remain inconsistent under biotic and abiotic stress, limiting their utilization in sustainable agriculture. There is an urgent need for cost-effective and multifaceted approaches to phytopathogens control, integrating bio-stimulants that enhance plant resistance and improve the biomarker of potato tuber quality. This study evaluated the efficacy of compost, macroalgae, *Trichoderma harzianum*, and arbuscular mycorrhizal (AM) fungi as bio-stimulants and their combinations in managing the black scurf disease of potato plants that causes serious yield losses.

**Results:**

The findings indicated that all assessed bio-stimulants markedly reduced the disease severity compared to the untreated control group. Notably, both *T. harzianum* and macroalgae demonstrated higher effectiveness when applied individually than other individual treatments, which achieved a reduction of DS by 71.57%, 69.61%, respectively, and DI by 71.43%, 64.28%, respectively. However, combinations of AM fungi (My) with macroalgae (Al), which achieved the highest reduction of DS by 83.46%, and DI (78.6%) in compared with the infested control. While the triple mixture of AM fungi, *T. harzianum*, and macroalgae exhibited superior efficacy in reducing disease incidence by 82.14% when compared to the infested control. Furthermore, all bio-stimulant treatments contributed positively to plant growth and tuber yield, particularly those involving AM fungi combined with macroalgae or their individual applications. The highest quality tubers of potato starch and –amylase content resulted from treatments with macroalgae alone or combined with mycorrhizal fungi. These tubers demonstrated improved tolerance to elevated temperatures at 60 °C in an oven until completely dry, with significant variations in potato quality correlating particularly with their starch and α-amylase contents. Furthermore, the influence of bio-stimulants on Indole-3-acetic acid, an important growth hormone, was consistent with observations obtained from greenhouse experiments.

**Conclusions:**

These findings highlight the potential of biologically-based strategies for managing black scurf in organic potato cultivation. Bio-stimulants, especially mycorrhizae and macroalgae, offer a sustainable approach to enhancing plant health, suppressing disease, and improving tuber quality.

## Background

Potato (*Solanum tuberosum* L.) is a crucial vegetable crop in Egypt, with 178,608 hectares planted and producing 29.2031 tons/ha of tubers in 2020 [1]. Farmers supply the potato crops with water and nutrients to increase the economic value and the land’s intensive use, which has probably contributed to higher early potato yields in recent decades [2, 3]. It is rich in carbohydrates, dietary fiber, proteins, vitamins, and minerals [4] as well as, it is an important source of antioxidants, especially phenolic compounds which are important for human health [5]. Their nutritional benefits make them a valuable addition to worldwide efforts to alleviate hunger [6].

Potatoes and other plants are susceptible to various pathogens that can severely impact yield and quality. *R. solani* influences root development and nutrient uptake; consequently, the plant’s biochemical pathways and enzymatic activity are affected [7, 8]. However, it is susceptible to fungal diseases like black scurf caused by *Rhizoctonia solani* (Telomorph: *Thanatephorus cucumeris* (Frank) Donk). Under a broad range of temperatures between 10 and 24 °C, *R. solani* is a common fungus damaging various crops [9]. The fungus develops in a wide temperature range and produces sclerotia on potato skin [10]. According to Zachow et al. [11], the sclerotial structures of *R. solani* in the soil are so persistent and permanent that they make the disease difficult to control, leading to poor-quality tubers and yield reduction [12]. The occurrence of black scurf in potatoes may vary based on the specific stage of growth, which is influenced by environmental factors and the inherent vulnerability of the potato type [13].

Effective management of *R. solani* requires environmentally sustainable strategies that compete with conventional fungicides. Biological control, using nonpathogenic antagonists, offers a safer approach to reducing disease risks, with natural agents emerging as a preferred method [14]. However, accurate disease management remains challenging. Applying various crop protection strategies can help mitigate disease severity, such as crop rotation, induced resistance, resistant cultivars, biological control, and soil solarization [15–17]. One effective approach is crop rotation with resistant cultivars over a three- to five-year period, which can reduce the incidence and severity of *R. solani*. However, El Bakali and Martin [18] note that small cultivation zones for potatoes complicate the process of crop rotation, necessitating innovative strategies to overcome such limitations. The term “bio-stimulant” was first defined in the scientific literature by Kauffman et al. [19], which promotes plant growth when applied in low quantities. Recently, bio-stimulants were defined as “a substance or microorganism that is applied to seeds, plants, or on the rhizosphere that stimulates natural processes to enhance nutrient uptake, nutrient use efficiency, tolerance to abiotic stress, or crop quality and yield” [20]. In the management of plant development, indole content is quite valuable. They boost root and fruit development as well as the immune system of the plant against biotic and abiotic agents damaging it [21]. Studies have shown that bio-stimulants such as humic substances, seaweed extracts, and amino acids enhance tuber size, shape regularity. Additionally, biostimulants improve nutrient uptake efficiency and promote enzymatic activity [14, 22, 23]. Research also indicates that foliar application of seaweed extract significantly increases tuber yield and carbohydrate content, demonstrating their role in improving crop productivity [24].

Previous research on bio-stimulants has primarily concentrated on their influence on plant growth. However, this scientific study aims to extend that understanding by analyzing the bio-stimulant’s effect, by assessing indole levels [22, 23]. Additionally, evaluating the effects of both individual and combined applications of the tested bio-stimulants, along with their synergistic interactions, would provide valuable insights into their impact on plant growth. Moreover, we will investigate how these bio-stimulants impact starch content, a critical factor for tuber quality. Specifically, our objective is to determine whether these substances can enhance the defense mechanisms of potato plants against *Rhizoctonia solani*, while simultaneously maintaining essential quality parameters. To achieve these goals, we will quantify and evaluate various enzymes and photochemical compounds associated with these processes.

## Methods


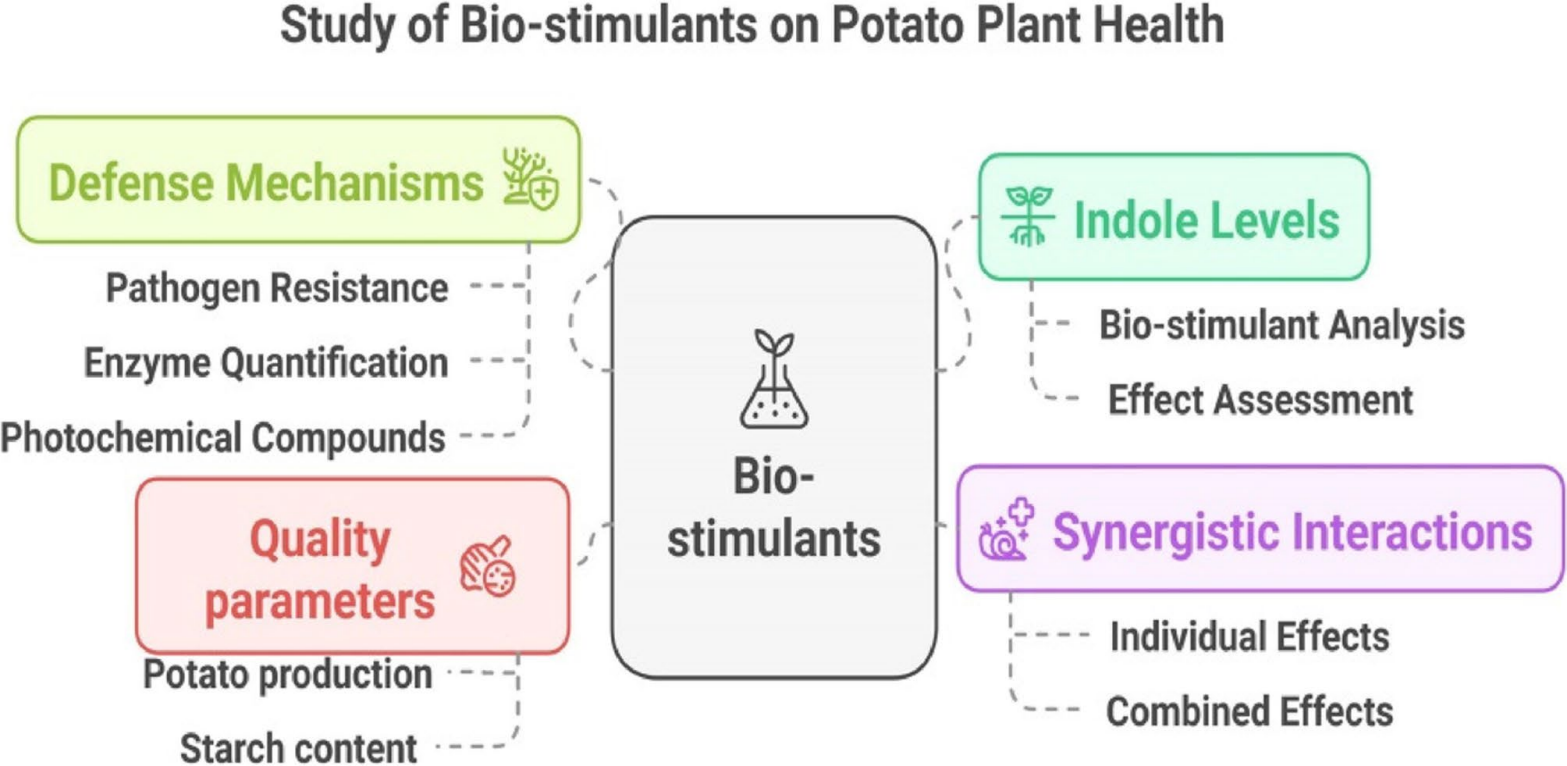


### Chemicals and other resources

0.1 M potassium phosphate buffer, pH 7.0. blank solution of 0.2 mL of McIlvaine buffer (pH 7.0) and 0.6 mL of dinitro salicylic acid (DNS) solution, comprising (1% DNS acid, 1% sodium hydroxide, 0.2% phenol, and 0.05% sodium sulfite),

2: 1.5: 6 g/Kg^− 1^ soil of NPK (Nitrogen: phosphorus: potassium).

Source of potato tubers: Healthy-looking tubers cv. *Spunta* with 3–4 eyes was obtained from the Potato Brown Rot Project (PBRP), Agricultural Research Centre (ARC). Giza, Egypt.

Source of Bio-stimulants: The Bio-stimulants organisms were obtained from Mycology and Disease Survey Research Department, Plant Pathology Research Institute, Agricultural Research Center, Accession number for Mycorrhiza (*Entrophospora etunicata*MT012422) [25] and *Trichoderma harzianum* (OL454813) [26] were maintained on suitable media at 25 ± 2 ˚C. Macroalgae (*Ulva fasciata*) were collected from the Mediterranean Sea following [27].

Source of Pathogen: The pathogen *Rhizoctonia solani* isolate has the Accession number AG-3 (OK275407) was obtained from the Vegetable Diseases Research Department, Plant Pathology Research Institute, ARC, Egypt.

### Pot experiments

#### Preparation of inoculum

In 500 mL sterilized glass flasks, *R. solani* inoculum was prepared. Each flask contained a 2:1 v/v mix of sorghum grains and sand, mixed, wetted with tap water, and autoclaved at 121 °C for 30 min. A 5-day-old *R. solani* 0.5 cm mycelial disc was inserted into the flasks. Flasks were kept at 24 ± 2 °C for 15 days based on Abd El-Aziz et al. [23].

To obtain the pots and soil prepared, the pot experiments were implemented over two summer seasons. A 5% formalin solution was used to sterilize a soil mixture, (that was composed of peat moss, clay, and sand in a 2:1:1w/w/w ratio.), and 30 cm X 25 cm diameter plastic pots (5Kg soil) for a suitable time to sterilize both of them, and then they were left to air dry for 24 h for pots and 2 weeks to the soil [28].

The physio-chemical characteristics of the soil EC (1.4 ds. m^− 1^, organic matter 1.8%, Anions [HCO3- (1.42 ml/l), Cl- (11.93 ml/l), SO4-2 (17.75 ml/l)], Cations [Ca + 2 (16.1 ml/l), Mg + 2 (4.33 ml/l), Na+ (4.7 m/l), K+ (0.2 ml/l)], Macro elements N (117.0 ppm), P (8.3 ppm), K (67.0 ppm), Macro elements Cu (0.0 ppm), Mn (0.3 ppm), Zn (0.13 ppm), Fe (0.6 ppm).

The pots were then filled in with sterilized soil and inoculated with the inoculum at the rate of 20 g/kg soil and water for five days before being treated. Pots of control were supplied with free *R. solani* sorghum grains and sand. Then Pots were treated with bio-stimulants individually and or in combination. Every treatment included five replicates, with each group of three pots serving as a single unit representing a replication. Each plastic pot contained one potato tuber, and the tubers were irrigated and fertilized as required by 2: 1.5: 6 g/Kg^− 1^ soil of a NPK (Nitrogen: phosphorus: potassium) according to [29].

#### The soil was treated with four bio-stimulants as follows

Arbuscular Mycorrhizal fungi *Entrophospora etunicata* (MT012422) were multiplied on Sudan grass (*Sorghum bicolor* (L.) Moench) grown in the Mycorrhizal greenhouse of “The Mycology and Disease Survey Department” for 16 weeks. The colonized Sudan grass roots (colonization rate about 90–100% of fine roots) were used as an inoculum (25 g inoculum/3 kg soil containing approximately 200 spores/g) was placed in the pot at 15 cm depth, before planting.

Compost was mixed into pots at a rate of 20% (v/v), and the mixer was analyzed for its physical, chemical, and biological properties at Water & Environment Res. Ins., ARC. Giza, as shown in Table 1.

**Table 1:** Physical, chemical, and biological properties of the tested compost

Characters	Unit	Mixed
Bulk Density	kg/m^3^	703
Moisture content	%	20
PpH	ds/m	7.5
EC	ds/m	5.65
NH_4_	Ppm	23
NO_3_	Ppm	920
Organic matter	%	36.91
organic carbon	%	19.5
C/N ratio	%	22.21
Total nitrogen	%	0.75
Total phosphorus	%	0.76
Total potassium	%	1.32
Bacteria and fungi	n.d.	n.d.
Nematodes	n.d.	n.d.

*T*. *harzianum* spores suspension (10^6^ spore/mL) was prepared at the rate of 5 mL/kg soil [30]. And macroalgae Ulva (Sea lettuce, *U. lactuca*) at the rate of 2 g dry powder/pot. Combinations of the above-mentioned treatments were added to the soil directly before the cultivation of tubers in pots and then irrigated and left for four days before the cultivation of the tubers at the rate of one tuber/pot. All treatments and their combination are presented in Table 2. In Fungicide treatment the soil was treated with Rizolex-T (50%WP) fungicide at the rate of 0.5 g/L water, while tubers were treated at the recommended dose (3 g/Kg tuber) during potato cultivation, and control treatments were " infested with *R. solani* both infested and un-infested by *R. solani*, were used as controls. Potato tubers were transplanted in each pot, providing five replicates for each treatment.

**Table 2:** Experimental design

No.	Treatments*
1	Mycorrhizae (My)
2	Compost (C)
3	*Trichoderma harzianum* (T.h)
4	Macroalgae (Al)
5	Mycorrhizae + Compost (My + C)
6	Mycorrhizae + *T. harzianum* (My + T.h)
7	Mycorrhizae + Macroalgae (My + Al)
8	Macroalgae + T. *harzianum* (Al + T.h)
9	Compost + T.h (C + T.h)
10	Compost + Macroalgae (C + Al)
11	Mycorrhizae + Compost + T. *harzianum* (My + C + T.h)
12	Mycorrhizae + Compost + Macroalgae(My + C + Al)
13	Mycorrhizae + *T. harzianum* + Macroalgae (My + T.h + Al)
14	Compost + Macroalgae + *T. harzianum* (C + Al + T.h)
15	Mycorrhizae + Compost + Macroalgae + *T. harzianum* (My + C + T.h + Al)
16	Fungicide treatment (Rizolex)
17	Infested control **
18	Untreated control ***

All treatments*: treated potato plants with different bio-stimulant treatments and grown in infested soil with* Rhizoctonia solani*, except Infested control **: un-treated potato plants grown in infested soil with* Rhizoctonia solani, *Untreated control***: un-treated potato plants grown in uninfested soil

### Experimental design

This experiment was carried out using a completely randomized block design with five replicates, each replicate containing three pots, and the treatments were detailed in Table 2.

#### Disease assessment

At ninety days post-planting, potato tubers were harvested and kept for four days under normal room conditions. Then the tubers were carefully removed residues of soil with water. Subsequently, the disease severity (DS) and disease incidence (DI) were assessed on ten tubers/replicate, which were randomly selected. A scale developed by Rauf et al. [31] was used to estimate the Disease severity, “DS: with a score of (0), no sclerotia are present. (1) less than 1% tuber area affected; A score of 10% or meaning little tuber area impacted (2). A score of 11–20% indicates moderate affected (3). A score of 4 indicates 21–50 tuber area affected. A score of 5 >51% indicates severe tuber area affected” before estimating DI and DS using the following formula:

$$\:DI\:\%\:=\left(\frac{Number\:of\:infected\:tubers}{Total\:number\:of\:tubers}\right)\times\:100$$

$$\:DS\%\:=\:\sum\:\frac{(No.\:of\:infected\:tubers\:X\:No.\:of\:scale)}{(Total\:No.\:of\:tubers\:\times\:highest\:No.\:of\:scale)}X100$$

$$\:Efficacy\%=\frac{Control-Treatment}{Control}\:\times\:100$$

### Growth parameters and yield assessment

Three plants of each treatment were randomly selected to estimate the growth parameters and yield i.e., Plant height (cm), stems number/plant, shoot fresh and dry weight, root fresh and dry weight (g)/plant, tuber number/plant, tuber weight (g), and weight 100 g of tubers after drying them in oven at 60 °C to calculate the total solids through moisture loss [32].

Chemical analyses provide valuable data to understand the effects of bio-stimulants and other management strategies on potato plants, plant health, stress responses, and disease interactions. By monitoring chlorophyll, amylase, and peroxidase levels.

### Defense-related enzyme peroxidase (PO) assessments

After 40 days of planting in potato leaves, the activity of PO was determined spectrophotometrically using a UV–Vis spectrophotometer (Uni-530 spectrophotometer) based on the method described by Kim and Yoo [33]. Plant material was homogenized at a ratio of 1:2 (W/V) with 0.1 M potassium phosphate buffer, pH 7.0. Extracts centrifuged at 12000xg, for 30 min at 4 °C. Enzyme activity is estimated at 470 nm. An enzyme’s activity is stated in units per milliliter of liquid (U/mL). One unit (U) is the enzyme’s ability to transform one µmole of the substrate into the product in one minute.

### Chlorophyll assessment

The Konica Minolta SPAD 502 was used to measure chlorophyll concentration 30 days after planting. The chlorophyll content of plant leaves can easily be determined using this portable, non-destructive meter to help assess plant vigor and stress tolerance [34].

### Starch assessment

Eight weeks after harvesting the tubers, potato starch and amylase content were evaluated in this study. The experiment predicted potato tuber starch using nondestructive near-infrared studies and chemometric models. Partial least squares and linear discriminant analysis were utilized, with the best accuracy achieved using the smoothing filter to out high-frequency noise from the spectral data Savitzky-Golay (S-G) and standard normal variant (SNV) preprocessed spectra. The results suggest nondestructive detection for internal quality prediction. To produce a cross-sectional slice of potato tubers, they were sliced around the center; an oven was used to dry the slices at 60 °C until completely dry. According to **Kim et al.** [35], the sample’s red, green, and blue (RGB) images were recorded. A neutral gray card was used to set the white balance. ImageJ software (NIH, Bethesda, MD, USA) was used to process the captured pictures. An RGB picture containing only a cross-section of the potato and excluding the background, then selected all areas of the potato slice, and it can be scanned images were turned into a histogram with the pixel count on the y-axis and grey intensity level (GIL) from 0 to 256 on the x-axis. Gil identified the content of starch employing A1: the smaller area where GIL >100, and A2: when starch is hydrolyzed, matched darker regions where GIL < 100. Finally, calculate Starch content according to this formula:

$$\mathrm{Starch}\;\mathrm{content}\;(\%)\;=\;\left[\left(\mathrm A1\div(\mathrm A1\;+\;\mathrm A2\;\right)\right]\;\times\;100$$

### Determination of α-amylase

Amylase activity relates to starch metabolism. Changes in amylase levels indicate alterations in starch content and quality. One gram of potato pieces, each with a thickness of 1 mm, was homogenized to obtain an enzyme extract, as described by Kim et al. [35]. The absorbance was measured at 540 nm with a blank solution of 0.2 mL of McIlvaine buffer (pH 7.0) and 0.6 mL of dinitro salicylic acid (DNS) solution, comprising (1% DNS acid, 1% sodium hydroxide, 0.2% phenol, and 0.05% sodium sulfite) according to Kim et al. [35].

### Indole-3-acetic acid (IAA) hormone assessment

One gram of potato plants, eight weeks after planting, was used to determine the concentration of IAA. The hormone was characterized at the Soil, Water & Environment Research Institute, Agriculture Research Center, Egypt, according to Raspor et al. [36].

### Statistical analysis

The Tukey Method and 95.0% Confidence were used to verify the statistical variations between treatment groups using the obtained data. All experiments were conducted in five replicates. To analyze the Data in Table four, one-way analysis of variance (ANOVA) was used to verify the statistical variations among treatment groups. And to determine if there was a statistically significant difference between the various groups. Treatment means were compared using the least significant difference (LSD) test at P-value < 0.05 with SPSS software v.8.0. All experiments were conducted using five replications. Then Principal Components Analysis (PCA) was run, as the first and second principal component scores were used to draw a biplot graph showing the best treatment for each trait.

## Results

### Growth parameters

**Fig. 1:**
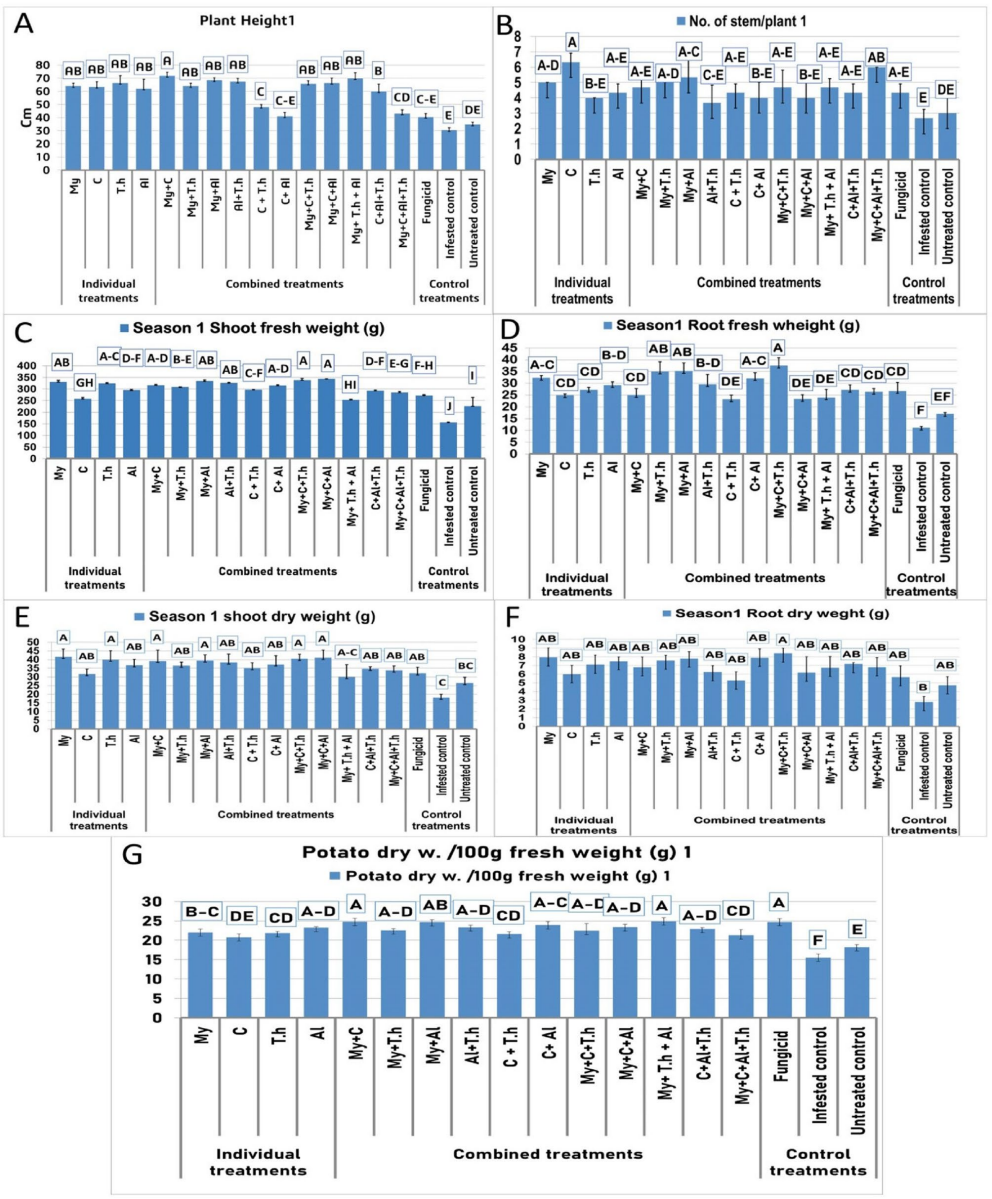
Effect of bio-stimulants on growth characteristics of potato plants inoculated with R. solani during season one. (Grouping Information Using Tukey Method and 95.0% Confidence for Growth parameters, Means that do not share a letter are significantly different)

**Fig. 2:**
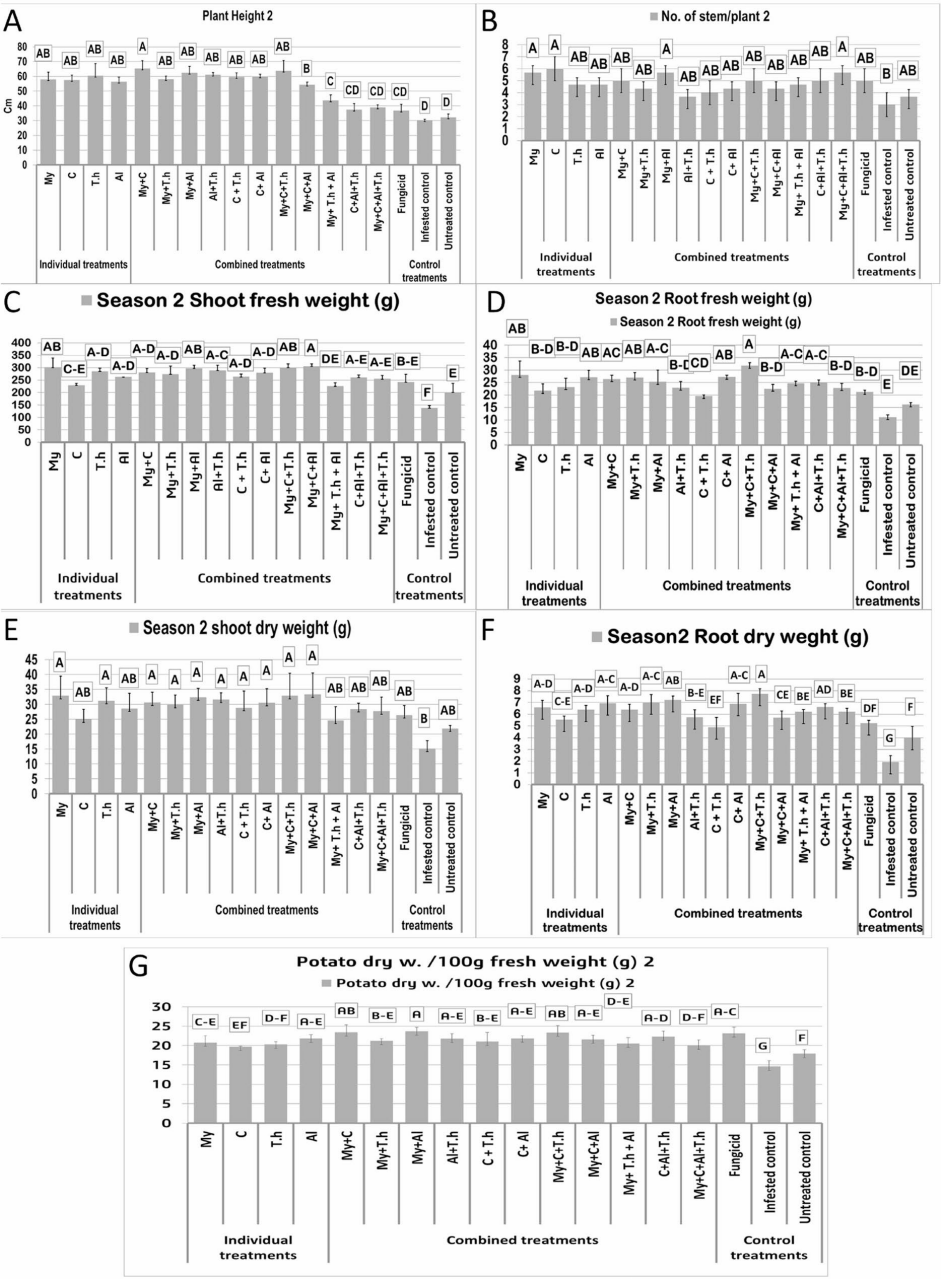
Effect of bio-stimulants on the growth characteristics of potato plants inoculated with R. solani during the second season. (Grouping Information Using Tukey Method and 95.0% Confidence for Growth parameters; Means that do not share a letter are significantly different)

**Fig. 3:**
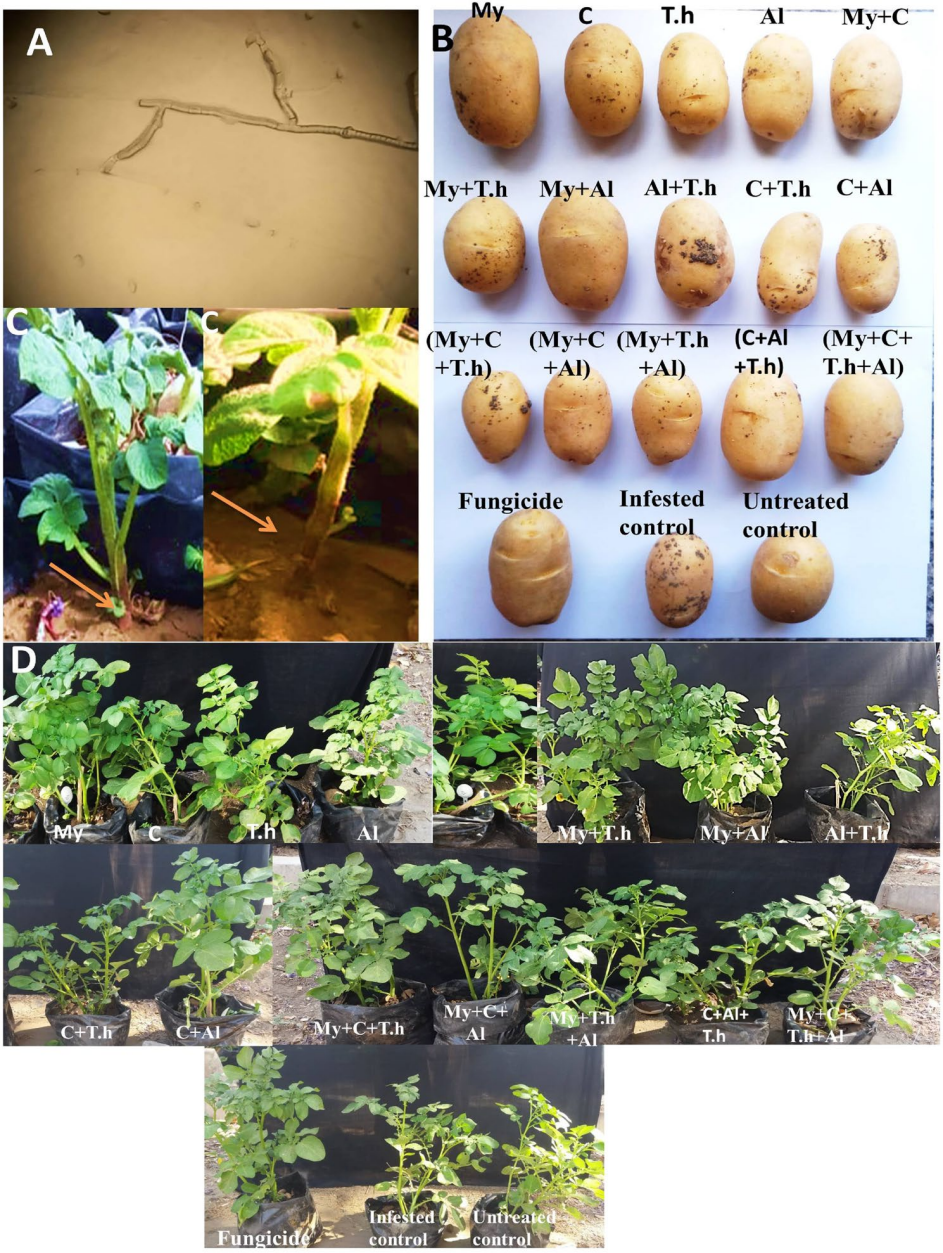
Effect of bio-stimulants on growth parameters and disease severity (DS) of potato plants infected with R. solani. **A**: mycelium of Rhizoctonia solani isolate. **B**: DS degrees on potato tubers after harvesting and incubation at room temperature. **C**: The symptoms of R. solani on potato plants. **D**: The impact of bio-stimulants on the development of potato plants treated with R. solan

The impact of bio-stimulants on the development of potato plants treated with *R. solani* was systematically evaluated through various growth parameters, including plant height (Fig. 1, 2 A) (Fig. 3), the number of stems per plant (Fig. 1,2B), shoot fresh weight (Fig. 1C), root fresh weight (Fig. 1, 2D), shoot dry weight (Fig. 1, 2E), root Dry weight (Fig. 1, 2 F), and potato dry weight/100 g fresh weight (Fig. 1, 2G). The results indicate that all bio-stimulant treatments led to an increase in the measured parameters. Notably, treatments incorporating mycorrhizae, whether used alone or combined with other treatments, yielded greater improvements across all growth measurements compared to the considered treatments. Specifically, mycorrhizal treatments and treatments integrating mycorrhizae with other bio-stimulants demonstrated a synergistic effect, further boosting main growth parameters resulting in a 85% increase in plant height (from 64.13 to 71.9 cm) compared to untreated control (34.83 cm), a 38.83% improvements in shoot fresh wheight (from 254.33 to 344 g) compared to untreated control (226.33 g), a 75.78% improvement in root biomass (from 23.3 to 37.53 g) compared to untreated control (16.99 g), and a 36.9% enhancement in overall yield (from 130 to 185.41 g) compared to untreated control (110.8 g). In contrast, applying compost alone resulted in the least increase in plant growth compared to other treatments. However, beneficial effects were observed when compost was combined with T.h, mycorrhizae, or macroalgae; this mixture significantly enhanced growth outcomes. In particular, the combination of compost and AM fungi demonstrated a positive influence on plant production, suggesting that compost may foster beneficial interactions between fungal symbionts and host plants. Nonetheless, it is important to note that while compost improved certain aspects of plant performance alongside mycorrhizal fungi applications, it also appeared to diminish both biomass production and antagonistic activity associated with *T. harzianum*, a biocontrol agent, which indicates a potentially adverse interaction. Additionally, neither the dual treatment involving C + Al nor the triple treatment encompassing My + C + Al produced significant enhancements in potato crop performance. These findings suggest that the nutrient composition of compost significantly influences interactions between soil microorganisms and host plants.

### Disease assessment

Season one data presented in Table 3 demonstrated the impact of various bio-stimulant treatments on DI and DS in potato plants growing in soil infested with *R. solani.* All examined bio-stimulant applications resulted in a reduction of both DI and DS values. However, the most effective individual treatments were identified as the T.h and Al, which achieved an impressive reduction of DS by 71.57%, 69.61, respectively, and DI by 71.43, 64.28%, respectively. Conversely, the least effective treatments involved mycorrhizae alone and compost alone, which resulted in reductions of DS at 66.5% and 64.7%, respectively, alongside DI reductions at 60.7% for mycorrhizae alone and 71.43% for compost alone. Regarding combined treatments, all mixtures enhanced plant health consistent with decreased levels of both DS and DI, particularly highlighting the effectiveness of the mycorrhizae + Macroalgae combination.

**Table 3:** Effect of bio-stimulants on disease incidence (DI) and disease severity (DS) of potato plants infected with R. solani (season 1 and season 2)

Treatments*	Season1				Season2			
	Disease severity (DS)		Disease incidence (DI)		Disease severity (DS)		Disease incidence (DI)	
	DS%	Efficacy	DI%	Efficacy	DS%	Efficacy	DI%	Efficacy
Individual treatments								
Mycorrhizae (My)	9.9 ^BC^	66.51	36.7 ^B^	60.71	11.3 ^BC^	63.9	26.7 ^B^	70.4
Compost (C)	10.4 ^BC^	64.76	26.7 ^B−D^	71.43	12.3 ^B^	60.5	23.3 ^B^	74.1
Trichoderma harzianum (T.h)	8.4 ^B−D^	71.57	26.7 ^B−D^	71.43	9.1 ^B−F^	70.8	26.7 ^B^	70.4
Macroalgae (Al)	8.9 ^B−D^	69.61	33.3 ^BC^	64.28	10.4 ^B−D^	66.5	16.7 ^BC^	81.5
Combined treatments								
My + C	7.4 ^C−E^	74.98	20.0 ^BD^	78.57	6.9 ^E−G^	78.0	33.3 ^B^	62.9
My + T.h	10.2 ^BC^	65.42	23.3 ^B−D^	75.00	9.6 ^B−E^	69.2	30.0 ^B^	66.7
My + Al	4.9 ^EF^	83.46	20.0 ^B−D^	78.57	5.9 ^F−H^	81.1	30.0 ^B^	66.7
Al + T.h	8.4 ^CD^	71.46	26.7 ^B−D^	71.43	7.8 ^D−G^	75.1	33.3 ^B^	62.9
C + T.h	10.9 ^B^	62.92	33.3 ^BC^	64.28	8.6 ^C−G^	72.5	23.3 ^B^	74.1
C + Al	7.9 ^B−D^	72.86	25.0 ^B−D^	73.21	5.9 ^F−H^	80.9	26.7 ^B^	70.4
My + C + T.h	9.3 ^B−D^	68.57	26.7 ^B−D^	71.43	5.4 ^GH^	82.6	23.3 ^B^	74.1
My + C + Al	6.6 ^D−E^	77.69	26.7 ^B−D^	71.43	7.6 ^D−G^	75.5	23.3 ^B^	74.1
My + T.h + Al	8.8 ^B−D^	69.96	16.7 ^C−E^	82.14	11.1 ^BC^	64.4	26.7 ^B^	70.4
C + Al + T.h	9.9 ^BC^	66.42	23.3 ^B−D^	75.00	8.6 ^C−G^	72.3	20.0 ^B^	77.8
My + C + Al + T.h	6.4 ^DE^	78.12	23.3 ^B−D^	75.00	6.22 ^FG^	80.0	23.3 ^B^	74.1
Control treatments								
Fungicid (Rizolex)	2.2 ^FG^	92.59	10.0 ^DE^	89.29	2.86 ^HI^	90.8	16.7 ^BC^	81.5
Infested control**	29.4 ^A^	0.00	93.3 ^A^	0.00	31.17 ^A^	0	90.0 ^A^	0
Untreated control***	0 ^G^	-	0 ^E^	-	0 ^I^	-	0 ^C^	-

All treatments*: treated potato plants with different bio-stimulant treatments and grown in infested soil with Rhizoctonia solani, except, Infested control **: un-treated potato plants grown in infested soil with Rhizoctonia solani, Untreated control***: un-treated potato plants grown in un-infested soil. Grouping Information Using Tukey Method and 95.0% Confidence for Growth parameters, Means that do not share a letter are significantly different

Promisingly, each bio-stimulant treatment significantly mitigated both DI and DS levels. The dual application of mycorrhizae and Macroalgae proved to be especially impactful, yielding significant reductions in both DS (83.5%) and DI (78.6%). Across seasons one and two, it was observed that singular applications such as mycorrhizae or compost exhibited lesser effectiveness in reducing DS and DI relative to the combination treatment. This observation may indicate that dual applications enhance plant resilience, but may not sufficiently induce systemic resistance against pathogens such as *R. solani.* The improved plant health associated with mixed treatments, including specifically mycorrhizae + Macroalgae is particularly noteworthy in both seasons; these combinations appeared more advantageous than individual applications when addressing control over *R. solani*. However, a triple mixture of AM fungi, compost, and Al exhibited superior efficacy in reducing disease incidence by 77.96% in season 1, and a triple mixture of AM fungi, T.h, and Al exhibited superior efficacy in reducing disease incidence by 82.14% in the second season. The results from season one are consistent with those recorded during subsequent assessments in the second season.

### Chemical parameter

**Fig. 4:**
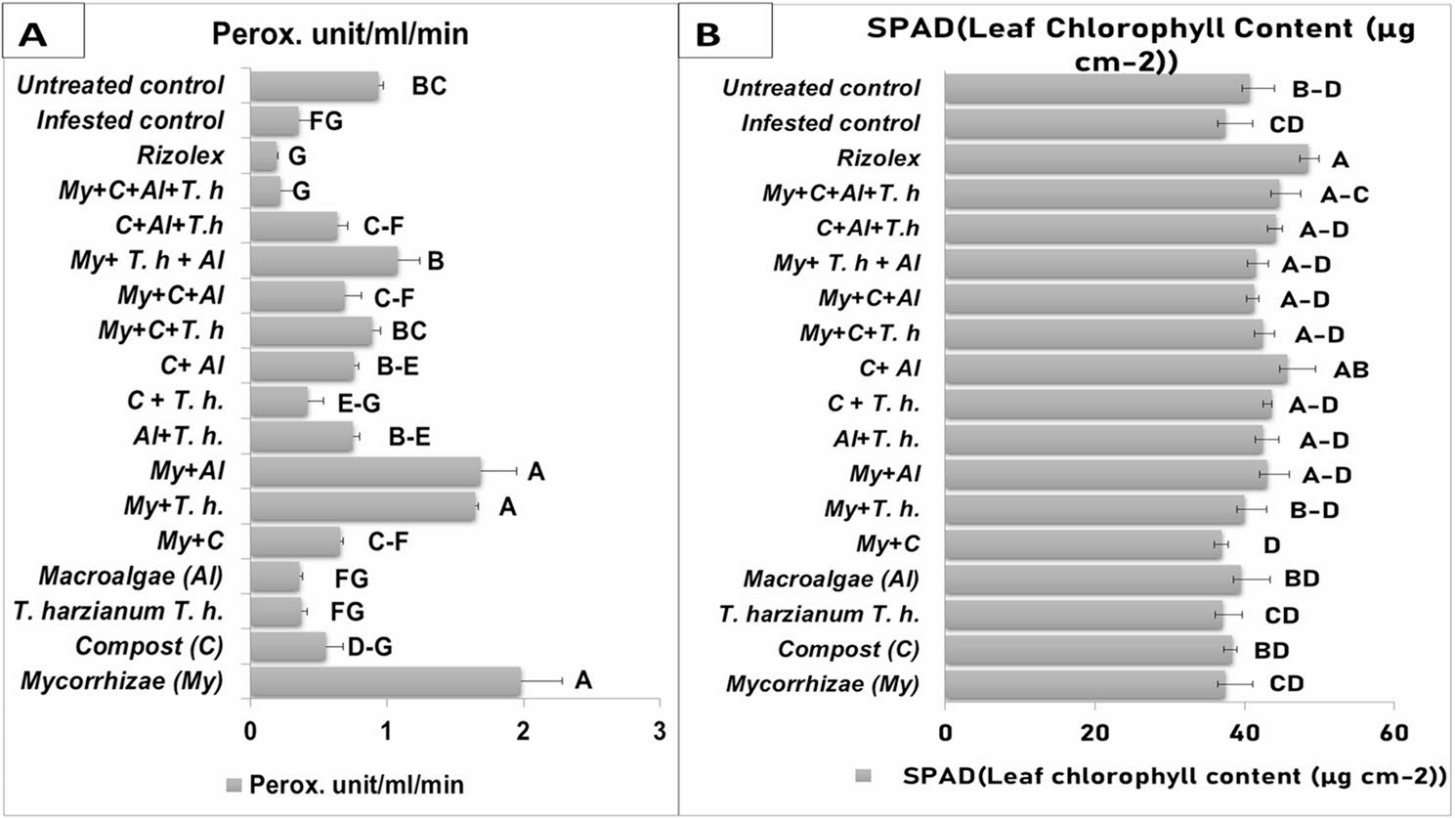
Effect of bio-stimulant treatments on (**A**) peroxidase enzyme activity and (**B**) total chlorophyll. (Grouping Information Using the Tukey Method and 95.0% Confidence for Peroxidase and Chlorophyll, means that do not share a letter are significantly different).

The Data presented in Fig. 4A indicate a marked increase in PO activity among plants treated with *R. solani*, particularly when bio-stimulants were applied. Notably, treatments that included mycorrhizae (My), and combinations of (My + *T. harzianum* (T.h)), (My + *Al*), and (My + *T. harzianum* + Macroalgae (Al)) demonstrated enhanced enzyme activity. Meanwhile, treatments consisting solely of *T. harzianum* (T.h), Macroalgae (Al), Compost + T.h, and My + C + T.h + Al exhibited a decrease in PO levels. These findings suggest that all treatments, including mycorrhizal fungi, whether added alone or in combination with other components, effectively mitigate the pathogen’s impact on plant health through their interactions with host plants. Regarding the enzymatic activity, treatment of Mycorrhizal fungi alone or with other treatments (My, My + C, My + Th, My + Al, My + C + T.h, My + C + Al, My + T.h + Al) led to an increase in enzyme levels, which indicates the enhancement of disease resistance. Furthermore, data illustrated in (Fig. 4B) demonstrate improved total chlorophyll content across all plant samples treated with bio-stimulants, especially (Al, My + Th, My + Al, Al + T.h, C + T.h, C + Al, My + C + T.h, My + C + Al, My + T.h + Al, C + Al + T.h, My + C + Al + T.h). Notably, those receiving treatments like Macroalgae alone or mixed products containing macroalgae coupled alongside other treatments showed significant enhancements compared to controls treated only with Rhizolix or untreated specimens. While Mycorrhizae alone, T.h alone, compost, and mycorrhizae + Compost did not have a positive effect on total chlorophyll.

Impact of mycorrhizae treatments with different bio-stimulants on Indole levels in potato plants infected with *R. solani*

### Impact of mycorrhizae treatments with different bio-stimulants on indole levels in potato plants infected with *R. solani*

**Fig. 5:**
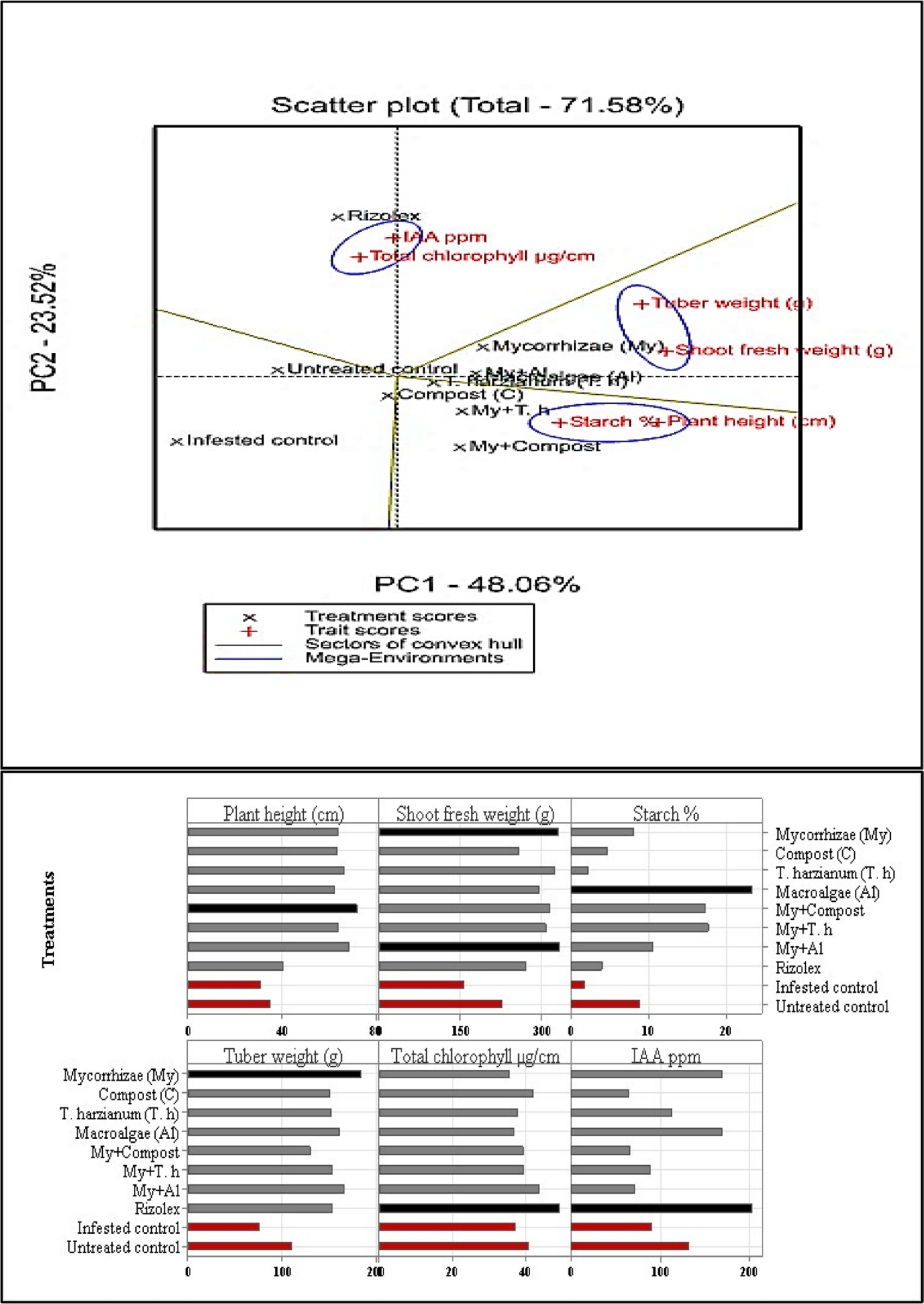
Principal component analysis (PCA) to evaluate Bio-stimulant treatments and their combination with mycorrhizae treatment on main parameters related to potato production and quality, and showing significant correlation among the studied variables: Plant height; shoot fresh weight; starch%; tuber weight; Total chlorophyll; IAA

Both Fig. 5 and Table 4 compare the effect of various bio-stimulants on IAA levels in potato plants infected with *R. solani*. The current study indicates a reduction in IAA concentration (measured in mg/kg) in plants experiencing stress due to *R. solani* infection. The decrease occurred in the levels of IAA in treatment with My + Al by 71.71 mg/kg, and Compost by 68.15 mg/kg. An increase in the IAA levels was observed after infested soil with *R. solani* treated with *T. harzianum* by 114.88 mg/kg, Macroalgae by 168 mg/kg, and mycorrhizae by 169.87. However, in contrast to experimental controls, the levels of these IAA were observed after *R. solani* treatment only (infected control) by 90.29 mg/kg, or infected plants treated with fungicide (Rizolex) by 202.36. However, in control without any treatment Indole level was 132 mg/kg.

**Table 4:** Evaluation of bio-stimulant treatments and their combination with mycorrhizae treatment on main parameters related to potato production and quality

Treatments	Plant height (cm)	Shoot fresh weight (g)	Starch %	Tuber weight (g)	Total chlorophyll µg/cm	IAA ppm
Mycorrhizae (My)	64.13 cd	331.43 a	8.03 e	183.33 a	35.40 d	169.00 b
Compost (C)	63.48 cd	257.73 e	4.72 f	150.73 bc	42.02 bc	65.25 g
T. harzianum (T. h)	66.47 bc	324.05 ab	2.21 h	152.32 bc	37.70 cd	113.00 d
Macroalgae (Al)	62.18 d	295.45 d	23.21 a	161.02 ab	36.88 cd	168.00 b
My + Compost	71.90 a	316.63 bc	17.22 b	130.20 cd	39.38 bcd	66.65 g
My + T. h	64.13 cd	308.28 cd	17.58 b	153.63 bc	39.48 bcd	89.25 e
My + Al	68.60 ab	334.30 a	10.50 c	166.55 ab	43.69 ab	71.28 f
Rizolex	40.58 e	272.21 e	4.02 g	153.6 bc	49.40 a	202.50 a
Infested control	30.60 g	156.07 g	1.69 h	76.27 e	37.01 cd	89.75 e
Untreated control	34.83 f	226.33 f	8.82 d	109.55 d	40.67 bcd	131.50 c

One-way analysis of variance (ANOVA) was used to verify the statistical variations among treatment groups. Treatment means were compared using the least significant difference (LSD) test at P-value < 0.05

### Assessment of treatment effects and their synergistic impact with mycorrhizae on potato production quality

The impact of bio-stimulants on the quality parameters of potato production has been thoroughly evaluated; focusing on aspects such as plant height, shoot fresh weight, starch content, tuber weight, total chlorophyll levels, and IAA concentration in potato plants. These comparisons aim to optimize potato yield while simultaneously reducing the prevalence of *R. solani*. Data presented in Table 4 and Table 5 demonstrates a significant influence of fungicide treatments on overall potato production and various growth metrics (as outlined in Fig. 5). Notably, mycorrhizal treatment followed by macroalgae applications positively impacted all assessed parameters. Additionally, tuber yields for plants treated with mycorrhizae alone or in combination with Macroalgae were markedly improved; these groups also showed enhanced starch content and IAA levels comparable to those observed with fungicide application. Furthermore, our findings indicate that leveraging a combination of bio-stimulants, specifically merging mycorrhizal treatment with macroalgae, can substantially elevate both tuber yield and starch content to levels akin to those achieved through traditional fungicide treatments. However, it is important to note that current data suggest no relation between IAA concentrations and the examined growth parameters.

**Table 5:** Evaluation of the effects of bio-stimulant treatments on potato plant productivity

Treatments	No. of tubers/Plant		Tuber fresh weight/Plant (g)	
	Season 1	Season2	Season 1	Season 2
Individual treatments				
Mycorrhizae (My)	11.00 ^A^	10.33 ^A^	185.41 ^A^	168.76 ^A^
Compost (C)	9.00 ^AB^	7.33 ^AB^	152.47 ^AB^	138.72 ^ABCD^
*Trichoderma harzianum* (T.h)	10.00 ^A^	9.33 ^AB^	154.07 ^AB^	140.20 ^ABCD^
Macroalgae (Al)	10.00 ^A^	9.00 ^AB^	162.87 ^AB^	148.19 ^ABC^
Combined treatments				
My + C	8.00 ^AB^	7.33 ^AB^	131.70 ^ABC^	119.86 ^BCD^
My + T.h	10.33 ^A^	9.00 ^AB^	155.40 ^AB^	141.34 ^ABCD^
My + Al	10.33 ^A^	9.33 ^AB^	168.47 ^AB^	153.23 ^ABC^
Al + T.h	11.33 ^A^	10.67 ^A^	180.06 ^A^	163.77 ^AB^
C + T.h	11.00 ^A^	10.00 ^AB^	169.13 ^AB^	153.89 ^ABC^
C + Al	7.67 ^AB^	8.33 ^AB^	124.83 ^ABC^	113.47 ^CDE^
My + C + T. h	10.67 ^A^	9.67 ^AB^	162.13 ^AB^	147.58 ^ABC^
My + C + Al	8.00 ^AB^	7.00 ^AB^	130.07 ^ABC^	118.23 ^CD^
My + T. h + Al	8.33 ^AB^	8.00 ^AB^	134.03 ^ABC^	122.05 ^BCD^
C + Al + T.h	8.33 ^AB^	8.00 ^AB^	142.63 ^AB^	129.78 ^ABCD^
My + C + Al + T. h	9.00 ^AB^	8.67 ^AB^	146.47 ^AB^	133.21 ^ABCD^
Control treatments				
Rizolex	9.33 ^AB^	9.67 ^AB^	155.37 ^AB^	141.38 ^ABCD^
Infested control*	5.00 ^B^	5.33 ^AB^	77.13 ^C^	70.22 ^E^
Untreated control**	7.00 ^AB^	6.33 ^AB^	110.80 ^BC^	100.80 ^DE^

All treatments*: treated potato plants with different bio-stimulant treatments and grown in infested soil with Rhizoctoniasolani, except Infested control **: un-treated potato plants grown in infested soil with Rhizoctonia solani, Untreatedcontrol***: un-treated potato plants grown in un-infested soil. Grouping Information Using Tukey Method and 95.0% 395Confidence for Growth parameters, Means that do not share a letter are significantly different.

Figure 5 analyzes the data using PCA in the form of a wagon wheel chart. 71.58% of the overall variation may be attributed to the combined contributions of factors PC1 and PC2. There is a cluster of growth and yield characteristics that can be found in the right quadrant. These characteristics were adversely affected by biotic stress. However, they showed signs of improvement when treatments including Mycorrhizae and Mycorrhizae + Macroalgae (My + Al)were applied. Conversely, a quality marker such as starch and plant height was grouped in another quadrant, these markers increase under biotic stress when soil amendments like Mycorrhizae + *T. harzianum* (My + T.h) and Mycorrhizae + Compost (My + C)are applied. Total chlorophyll and IAA are also clustered together, exhibiting heightened activity under chemical control (Rizolex) and further enhancement with the applied treatments than the infested control. The growth attributes, including shoot fresh weight, plant height, starch%, and yield (tuber weight), show positive correlations with disease reduction efficacy in Table 3. This suggests that reducing the disease’s aggressiveness through the applied treatments enhances the growth, and photosynthesis, as well as yield parameters of potato plants Table 5. Similarly, Table 4 also reveals positive correlations among these variables.

### Starch content evaluation

**Fig. 6:**
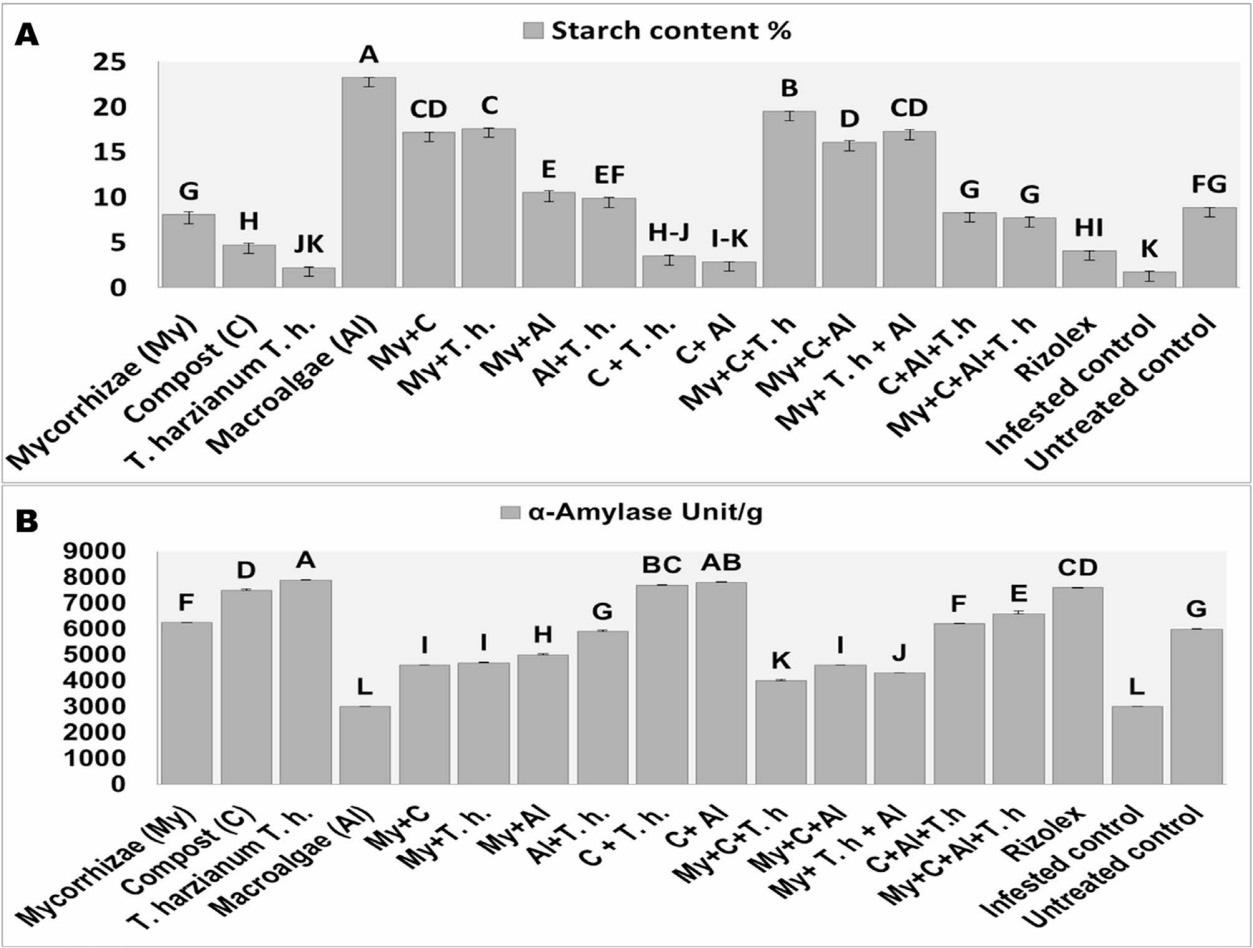
Starch (**A**) and α-amylase contents (**B**) of potato tuber treated with different 410 bio-stimulants under Rhizoctonia solani bio-stress. (Grouping Information Using the 411 Tukey Method and 95.0% Confidence for Starch and α-amylase contents, Means that do 412 not share a letter are significantly different)

**Fig. 7:**
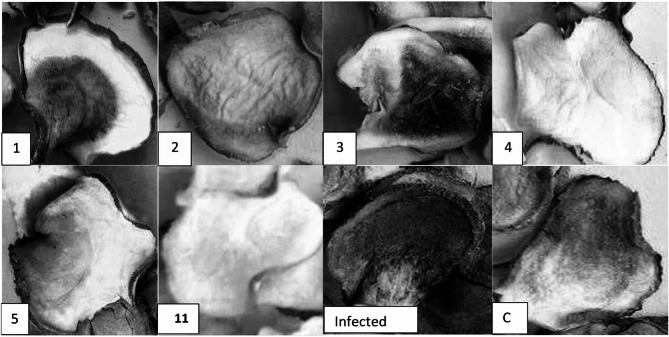
Cross-sections of potato tubers from plants treated with individual or in combination of bio-stimulants (mycorrhizae (1), compost (2), Trichoderma harzianum (3), Macroalgae (4), Mycorrhiza+compost (5), and Mycorrhizae+Compost+T. harzianum (11)), referring to degrees of incomplete starch degradation compared with infested treatments with Rhizoctonia solani and un-treated control (C)

Data presented in Fig. 6 indicate that the starch content (Fig. 6A) and α-amylase levels (Fig. 6B) of potato tubers significantly influence their physicochemical properties associated with storage and thermal preparation. This study assessed the changes in the starch and α-amylase content eight weeks post-harvest. The determination of starch content was executed using the partial least squares (PLS) method to quantify the starch content, reflecting changes in internal quality based on moisture, starch, and α-amylase levels. Figures 7 illustrates the cross-section analysis of potato tubers according to the degree of starch decomposition. As shown in Figure 6B, treatment groups involving Compost, *T. harzianum*, and Mycorrhizae resulted in increased α-amylase activity. Conversely, a significant decrease in α-amylase activity was observed following treatment with Macroalgae, suggesting an associated increase in starch content.

## Discussion

### Growth parameters

The current study highlights the complex interactions among various bio-stimulants, compost, AM Mycorrhizal fungi, *T. harzianum*, and Macroalgae in potato cultivation. This research specifically assessed the effects of these bio-stimulants on potato plants under *R. solani* infection. Particularly, bio-stimulants that included mycorrhizae demonstrated a significant capacity to enhance plant growth; conversely, compost applied independently proved ineffective. However, when compost was combined with either mycorrhizae or macroalgae, its negative effects were significantly alleviated. Supporting recent research indicates that utilizing a combination approach involving arbuscular mycorrhizal fungi (AMF) alongside Compost notably enhances both vegetative growth outcomes as well as essential soil properties. So the incorporation of beneficial microorganisms into compost resulted in improved plant growth and yield [37]. It is important to note that the optimal dosage and application timing for bio-stimulants are contingent upon factors such as crop type, soil characteristics, and environmental conditions [38]. Additionally, the quality of compost is influenced by its source materials and the specific processes used during composting, both of which have direct implications for plant health. Variations in chemical composition, such as elevated nitrate levels coupled with low phosphorus content, can adversely affect plant growth outcomes [39]. The current investigation showed that the compost used in this study (Table 1) had high levels of NO3, carbon, and low levels of phosphorus. This chemical composition of compost may have negative effects on plant growth. But compost with Mycorrhizal fungi increased plant production, showing a beneficial effect on fungal symbionts. However, the compost reduced the biomass and antagonistic activity of *T. harzianum*, suggesting a negative impact of the compost on this biocontrol agent. Moreover, neither the dual mixture of (AL + C) nor the triple mixture of AL + C + MY has significant effects on potato plants. These results suggest that the nutrient composition of the compost influences the interactions between the soil microorganisms and their plant hosts. According to a recent study, the combined effect of arbuscular mycorrhizal fungi (AMF) and Compost improved growth and soil parameters [40]. Abdelhafez et al. [37] confirmed our findings when they studied the effects of different bioinoculants on common beans infected with *Sclerotium rolfsii*. The bioinoculants were found to improve the plant defense enzymes, survival rates, and nutrient uptake of the beans, mainly when combined. The compost may interfere with the beneficial effects of *T.h* and macroalgae. Some studies have reported that compost can reduce the colonization and activity of *T.h* on roots, affecting plant availability and uptake of nutrients; [22, 39].

### Disease index

All bio-stimulant treatments significantly reduced both DI and DS, highlighting their potential to enhance plant health and mitigate potato diseases. The combination of mycorrhizae and Macroalgae is the most effective treatment, reducing potato diseases. Bio-stimulants play a crucial role in disease management strategies. Mycorrhizae improve nutrient uptake, and Macroalgae stimulate plant defense responses. Treatments with mycorrhizae or compost alone are less effective. Combining mycorrhizae and Macroalgae can enhance plant health and manage pathogens like *R. solani*. Bio-stimulants, such as seaweed extracts and humic substances, promote plant growth and reduce reliance on conventional fertilizers [22]. *T. atroviride* has also shown considerable efficacy in suppressing *R. solani* growth [41]. Nonetheless, the anticipated positive impact on potato growth from combining T.h or Macroalgae with compost was not realized. This outcome may be attributed to nutrient absorption by T.h itself. These findings align with [42], who concluded that compost amendment reduces the beneficial effects of T.h on potato plants. Furthermore, existing research reveals that different strains of Trichoderma exhibit varying capacities to support plant development through mechanisms such as root colonization, antifungal metabolite production, resistance induction, and nutrient solubilization [43]. The combination of mycorrhizae and macroalgae showed strong potential in managing *R. solani*, but the molecular mechanisms behind their interactions were underexplored. Studies suggest that arbuscular mycorrhizal fungi enhance plant immunity and systemic resistance, while macroalgae can modulate defense pathways and activate salicylic acid and jasmonic acid pathways, which enhance plant defenses [44–46]. Ali et al. [47] recorded that macroalgae stimulate pathogen-associated molecular pattern (PAMP) recognition, inducing immune responses.

#### Chemical parameter

Peroxidase enzymes play a role in defense against oxidative stress. This enzyme has been used to indicate plant responses to stress-causing pathogens when peroxidase activity is high. The study found that peroxidase levels in plants increased significantly when treated with *R. solani* and bio-stimulants. Treatments with mycorrhiza, especially in combination with *T. harzianum* and Macroalgae, showed enhanced enzyme activity. Conversely, treatments without mycorrhizae exhibited decreased enzyme activity. Overall, the presence of mycorrhizal fungi, alone or in combination with other treatments, helped mitigate the negative effects of the pathogen on plants through synergistic interactions. Additionally, the use of mycorrhizal fungi led to improved disease resistance by increasing enzyme activity, particularly PO, which plays a crucial role in plant defense mechanisms against pathogens by oxidizing phenolic compounds. Enzymes such as polyphenol oxidase (PPO) and PO facilitate the oxidation of phenolic substrates; this process generates reactive oxygen species (ROS), quinones, and lignin. These compounds are associated with antimicrobial properties [48]. Existing literature underscores diverse roles and responses related to the interaction between mycorrhizae and pathogens within plant systems. In particular, PO plays a vital role in lignin synthesis, which is a structural phenolic polymer that fortifies cell walls and enhances resilience against both biotic and abiotic stressors. PPO is mainly involved in producing quinones, which are reactive compounds that can inhibit the growth of pathogens by cross-linking proteins or forming toxic complexes [49]. Mycorrhiza treatments can enhance phenolic defense capacity in plants exposed to *R. solani* by stimulating phenolic biosynthesis or activating latent phenoloxidases. Various factors influence PO and PPO activity in plants, with treatments like *T. harzianum* or Macroalgae potentially reducing the need for phenolic defense. Other defense mechanisms may be induced, such as the activation of defense genes, the production of antimicrobial compounds, the modulation of plant hormones, and the priming of plant immune responses [49].

Plants treated with bio-stimulants showed improved total chlorophyll levels, with certain combinations like macroalgae and compost + Macroalgae having significant effects. Mycorrhizae and *Trichoderma* alone did not positively affect chlorophyll levels, possibly due to increased organic matter promoting pathogen growth. Some studies suggest *Trichoderma* sp. can inhibit plant pathogens, but others found no significant increase in chlorophyll levels in tomato leaves treated with *Trichoderma* strains compared to controls [50]. *Trichoderma* sp. treatment reduced chlorophyll and increased flavonols in one cultivar but not in another due to altered gene expression and linked to lower nitrogen content [38]. *Trichoderma* sp. colonization changed the phenylpropanoid pathway in tomato plants, affecting chlorophyll content differently in different cultivars [38, 51]. On the other hand, Ikram et al. [52] found that *T. reesei* increased the total chlorophyll concentration of wheat plants exposed to salt stress. Another study found that *T. asperellum* improved the chlorophyll content of tomato plants [53]. While some studies reported positive effects of *Trichoderma* sp. on chlorophyll under stress conditions, others found negative effects on root growth and development [54]. One study found that some strains of *Trichoderma* spp. inhibited the expression of the auxin reporter gene DR5 in Arabidopsis primary roots, which affects root growth and development. Another study found that *T. asperellum* reduced the chlorophyll content of maize plants, which affects photosynthesis and biomass production [53]. *Trichoderma* sp. may also affect chlorophyll content depending on factors like strain, plant species, soil type, and environmental conditions. In conclusion, the impact of *Trichoderma* sp. on chlorophyll levels is not consistent and varies based on specific circumstances and interactions.

#### Biocontrol potential on indole levels, potato production quality, and starch content evaluation

The current study analyzed the impact of different treatments on indole levels in potato plants infected with *Rhizoctonia solani*. Infection stress may lead to increased levels of indole as a defense mechanism, with indole compounds like IAA playing a role in stress signaling. Bio-stimulants like mycorrhizae can help regulate hormone levels, potentially reducing indole levels and improving plant health. The fungicide “Rizolex” increased indole levels significantly, suggesting it may induce additional stress or hormonal changes. Beneficial microbes such as mycorrhizae and *T. harzianum* can interact with plant roots to manage stress, resulting in lower indole levels.

The current study also compared various quality parameters of potato plants to enhance production and reduce *Rhizoctonia solani* disease. Wang et al. [55] found that auxin plays a crucial role in arbuscular mycorrhizal symbioses, and promotes tomato growth in continuous cropping substrates. On the other hand, Macroalgae are known for their ability to absorb nutrients and heavy metals from soil. Evidence has emerged that some phytohormone signaling components of higher plants are present in microalgae. Seaweed extracts contain small amounts of natural auxins and indoles that help stimulate rooting, which, when combined with cytokinins, result in the production of a greater root mass that provides better uptake of water and minerals and a healthier, more stress-resistant plant. Seaweed extracts are particularly rich in cytokinins [56]. Seaweed extracts impact plant gene transcription levels, influencing growth hormone synthesis, like auxin, cytokinin, and ABA. Increased root mass improves moisture and nutrient uptake, leading to more foliage, flowers, and stress resistance. Compost effects on growth vary by type [57].

Starch and α-amylase contents of the potato tuber affected the physicochemical properties of potato storage and heat preparation. In this study, the starch and α-amylase content of potatoes was analyzed eight weeks post-harvest using the PLS method. Changes in potato quality post-harvest were assessed based on moisture, starch, and α-amylase levels. Treatments with Compost, *T. harzianum*, and Mycorrhizae increased α-amylase activity, while Macroalgae treatment decreased it, leading to higher starch content. Previous studies showed that AM colonization affects root starch levels, with starch accumulating before colonization in response to AM fungal signals. However, their results indicated that all varieties exhibited the highest levels of α-amylase activity at 3 weeks [58]. Hagenimana et al. [59] found that sweet potatoes’ glucose content increased and total starch decreased over time, impacting the paste viscosities of potato flour negatively. Contrary to gelatinization, retrogradation of potato starch is the process of disorder amorphous starch into order crystalline starch, which has short-term and long-term stages [60]. Commercial algae extracts, which contain a variety of bioactive substances, including growth regulators, vitamins, and minerals, are often included as bio-stimulants. Algae extracts are rich in several important macro- and microelements, as well as genes, amino acids, and plant hormones, including auxins and cytokinins. A diverse array of free amino acids, including aliphatic, aromatic, citric, and essential amino acids generated by enzymatic hydrolysis, are present in the bio-stimulant mix that is sourced from natural seaweed (Macroalgae) extract. In addition to these compounds, seaweed contains organic nitrogen, boron, magnesium, iron, and zinc [56, 61].

Finally, when comparing the effects of bio-stimulant treatments on disease severity in potato crops, a comparative analysis of disease suppression suggests that mycorrhizal and macroalgal applications, which achieved an efficacy of 83.46%, provide effective alternatives to traditional fungicides, which reached 92.59% efficacy with no significant differences between them. Additionally, Mycorrhizal treatment improved tuber yield by 19.3% than fungicide treatment, and starch content showed a significant increase than fungicide treatment. The highest starch content was recorded in Macroalgae (Al) treatment, followed by Mycorrhizae combined with compost and T. harzianum. These findings indicate that bio-stimulants can enhance crop productivity, with the combination of mycorrhizal treatment and macroalgae proving particularly beneficial, achieving good results comparable to fungicide applications (46,24).

## Conclusion

The findings of the study demonstrated the synergistic effects of mycorrhizae and macroalgae in enhancing tuber production, starch content, and plant health in potato plants, achieving results comparable to fungicide treatments. The combined treatment demonstrated superior efficacy over individual applications. Notably, tuber production in plants treated with mycorrhizae alone, macroalgae alone, and their combinations showed significant enhancement. These treatments also resulted in improved starch content and elevated levels of Indole-3-acetic acid, closely rivaling the effects observed with fungicide treatment.

## Data Availability

All data supporting the findings of this study are available within the paper and its Supplementary Information. HPLC analysis of indole in potato plants are available in Supplementary material Fig S2-Arbuscular Mycorrhizal fungi Entrophospora etunicata Accession number (MT012422) https://www.ncbi.nlm.nih.gov/nuccore/MT012422-Rhizoctonia solani AG-3 Accession number (OK275407) https://www.ncbi.nlm.nih.gov/nuccore/OK275407-Trichoderma harzianum Accession number (OL454813), https://www.ncbi.nlm.nih.gov/nuccore/OL454813.1/.

## Abbreviations

My: Mycorrhizae
C: Compost
T.h: *Trichoderma harzianum*
Al: Macro algae
My+C: Mycorrhizae+Compost
My+T.h: Mycorrhizae+*T. harzianum*
My+Al: Mycorrhizae+Macroalgae
Al+T.h: Macroalgae+*T. harzianum*
C+T.h: Compost+ *T. harzianum*
C+Al: Compost+ Macroalgae
My+C+T.h: Mycorrhizae+Compost+*T. harzianum*
My+C+Al: Mycorrhizae+Compost+macroalgae
My+T.h+Al: Mycorrhizae+*T. harzianum*+ Macroalgae
C+Al+T.h: Compost+Macroalgae+*T. harzianum*
My+C+T.h+Al: Mycorrhizae+Compost+macroalgae+*T. harzianum*
PBRP: Potato Brown Rot Project
ARC: Agricultural Research Centre
PPRI: Plant Pathology Research Institute
S-G: Savitzky-Golay
SNV: Standard normal variant
GIL: Grey Intensity Level
RGB: Red, green, and blue
PLS: Partial least squares
DI: Disease Incidence
DS: Disease Severity
PPO: Polyphenoloxidase
PO: Peroxidases
PCA: Principal Components Analysis
ABP1: Auxin Binding Protein 1
IAA: Indole-3- acetic acid
ABA: Abscisic acid
NBI: Nitrogen balance index
